# A Double-Blind, Placebo-Controlled Trial Demonstrating the Safety, Tolerability, and Pharmacokinetics of Single, Escalating Oral Doses of RTI-336

**DOI:** 10.3389/fphar.2018.00712

**Published:** 2018-07-10

**Authors:** F. Ivy Carroll, Thomas R. Kosten, Jeffrey J. Buda, Laurene Wang, Bradford B. Walters

**Affiliations:** ^1^RTI International, Research Triangle Park, NC, United States; ^2^Psychiatry, Neuroscience, Pharmacology and Immunology & Pathology, Baylor College of Medicine, Houston, TX, United States; ^3^INDApharma, Chapel Hill, NC, United States

**Keywords:** RTI-336, dopamine transporter inhibitor, Clinical Trials, cocaine dependence, addiction and addiction behaviors, pharmacokinetics and drug metabolism (PDM), safety, tolerability

## Abstract

**Background:** Preclinical and clinical data suggest that a compound which binds potently to and inhibits the dopamine transporter, but with a slower onset and offset rate than cocaine and with less abuse potential and psychomotor stimulant activity, could be a useful adjunct in the treatment of cocaine dependence.

**Methods:** We assessed the safety, tolerability, and pharmacokinetics (PK) of oral single doses (0.3, 1, 3, 6, 12, and 20 mg) of such an analog, RTI-336, in a randomized, double-blind, and placebo-controlled trial in healthy adult males. Pre-dose and post-dose safety assessments included physical examinations (including neurological examination); orthostatic vital signs; 6-lead continuous electrocardiogram (ECG) telemetry monitoring pre-dose to 8 h post-dose; 12-lead ECGs; clinical chemistry, hematology, and urinalysis; mini mental status examinations; and adverse events. RTI-336 PK was assessed in plasma and urine.

**Results:** 22 participants were enrolled. RTI-336 was well-tolerated up to the maximum evaluated dose of 20 mg. PK analyses demonstrated good absorption with peak plasma maximum concentrations (C_max_) occurring around 4 h post-dose and consistent half-lives of around 17 h for the 6, 12, and 20 mg doses. Plasma drug exposure and C_max_ increased in proportion to dose. Only 0.02% of RTI-336 excreted was unchanged in urine. Active metabolites UC-M5, UC-M8, and UC-M2 were measurable in plasma and urine, with plasma C_max_ of UC-M5 and UC-M8 exceeding that of RTI-336. Three AE possibly were related to RTI-336 and resolved with discontinuation; the one serious AE was unrelated to RTI-336. 2 participants had transient and mild serum total bilirubin elevations (<1.5× upper limit of normal) at day 3 post-dose which resolved by day 8 post-dose.

**Conclusion:** All doses including the highest (20 mg) showed excellent safety and tolerability, and further studies in humans are warranted.

**Trial Registration:** ClinicalTrials.gov Identifier: NCT00808119.

## Introduction

Cocaine abuse has been a public health threat in the United States since the mid- to late-1970s. This epidemic stabilized about 10 years ago, with the most recent (2014) National Survey on Drug Use and Health estimating that there were 1.5 million persons aged 12 or older who were then cocaine users, including 0.9 million with cocaine use disorder. The most recent (2011) Drug Abuse Warning Network data reported 505,224 drug-related emergency department visits for cocaine; that year there were 152,038 cocaine-related admissions to substance abuse treatment centers (Substance Abuse and Mental Health Services Administration, Center for Behavioral Health Statistics and Quality, 2011, 2015a,b).

As a physiological brain disorder cocaine dependence could benefit from pharmacotherapy, but no agents have shown consistent efficacy (Carroll et al., 1999; Howell and Wilcox, 2001; Newman and Kulkarni, 2002; Carrera et al., 2004; Shorter et al., 2014; Andry et al., 2016). A wide range of mechanisms of action have been investigated and failed as treatments, including agents both for raising and lowering the activity of dopamine (bromocriptine vs. aripiprazole), norepinephrine (methylphenidate vs. doxazosin), and serotonin (serotonin reuptake inhibitors vs. ondansetron) systems. Glutamate and gamma-aminobutyric acid modulators and blockers have also been tested and failed. More exotic approaches include vaccines, enzymes, and opiate partial mu agonist/kappa antagonists like buprenorphine. None of the over 50 agents tested has shown efficacy in large-scale, placebo-controlled, multisite studies, and none have obtained US Food and Drug Administration (FDA) approval for marketing.

Development of a new medication to treat cocaine addiction would be a major medical and social breakthrough. Since human neuroimaging studies find a significant correlation between dopamine transporter (DAT) occupancy and the subjective high from cocaine (Volkow et al., 1997), one approach has been to consider a compound that will bind selectively to the DAT (Ritz et al., 1987; Bergman et al., 1989; Kuhar et al., 1991; Wise et al., 1995; Wilcox et al., 2000). As reviewed recently, reductions in cocaine consumption during agonist-medication maintenance can be dramatic, and agonist medications, such as amphetamine produce surprisingly weak evidence for abuse potential or cardiovascular risk in cocaine abusers (Negus and Henningfield, 2015). However, regardless of evidence for therapeutic effectiveness, it is unlikely that an agonist treatment like amphetamine will be FDA approved because its abuse, diversion, and toxicities are of great concern in the United States. Important considerations for such an agonist pharmacotherapy are that it should have a slower onset rate and an extended duration of action relative to cocaine, should not produce sensitization and should have less abuse potential than cocaine or amphetamine (Samaha and Robinson, 2005; Kimmel et al., 2007).

In this paper, we report the results of the first clinical trial to study RTI-336 in humans. RTI-336 is a 3-phenyltropane analog that is a DAT selective inhibitor possessing suitable pharmacological properties and that suppresses cocaine self-administration in rat and rhesus monkey models (Carroll et al., 2006a,b; Carroll, 2007; Howell et al., 2007). Because RTI-336 mimics the DAT-binding properties of cocaine but is less stimulating and acts with a slower receptor onset and offset, it may prove useful as treatment for cocaine dependence that can mitigate concerns about abuse liability of DAT transporter inhibitors as treatments (Howell and Negus, 2014). Trial RTI-336-001 evaluated safety, tolerability, and pharmacokinetics (PK) following the administration of single, oral doses of RTl-336, with the possibility of identifying the maximum tolerated dose in humans.

## Participants and Methods

### Study Design

RTI-336-001 was a single-center, double-blind, placebo-controlled, and randomized clinical trial to evaluate the safety, tolerability, and PK of single, escalating oral doses of RTI-336 (0.3, 1, 3, 6, 12, and 20 mg) in 2 cohorts (lowest 3 doses and highest 3 doses) of healthy male participants. RTI-336 and placebo capsules were identical in appearance, and participants, care providers, and those assessing outcomes were blinded to study drug assignment. Each cohort was to be enrolled in 2 groups of 5 participants (randomized 4:1 drug:placebo independently at each dose); the second group was dosed 24 h after the first group, following a safety review of the first group. Each participant would receive up to 3 single, escalating oral doses (in periods 1, 2, and 3) of study drug under fasting conditions, with a minimum of 10 days between doses, and could only participate in one cohort. The number of participants is typical for first-in-man safety and PK studies, and the trial ran to completion.

Subject randomization was performed by an RTI statistician (blinded to specific participants) using standard SAS protocols. Each cohort was randomized in two blocks of three to five participants (depending on the final number eligible on the morning of dosing, always maintaining one placebo dose). The statistician provided the information to the unblinded pharmacist (the only unblinded person at the site); the documentation was kept locked in the pharmacy, itself with highly controlled and limited access. Immediately prior to dosing, the pharmacist prepared individually packaged and labeled doses as assigned. These subsequently were transported from the pharmacy into the clinic and administered to the participants by blinded site personnel. Prior to a dose increase within a cohort, all safety and PK data were reviewed by a Safety Review Team as guided by a Data and Safety Monitoring Plan (DSMP); data from cohort 1 were submitted to the FDA prior to initiating cohort 2. The DSMP included clinical and laboratory criteria for both dose reduction and study termination, and no subject met these criteria during this study. In each of the 3 dose periods, participants were admitted to the clinic the night before dosing (day 0) for urine drug and alcohol testing and were to be confined to the clinic for 3 nights.

This study was carried out in accordance with the International Conference on Harmonisation E6 Consolidated Guidance for Good Clinical Practice (1996) and the US Code of Federal Regulations 21 parts 50 and 56. The protocol was approved by the Institutional Review Board at the clinical site, Comprehensive Phase One in Miramar, FL, and is reported at ClinicalTrials.gov (NCT00808119). All subjects gave written consent in accordance with the Declaration of Helsinki (as amended in 1996).

### Participants

#### Inclusion Criteria

Participants were healthy males, 18–50 years old, ≥50 kg weight, and body mass index (BMI) 18–30 kg/m^2^, with a negative pre-study drug screen, no history of use of illicit drugs within 12 months of the screening visit or other substances of abuse within 12 months of the screening visit, no tobacco use for ≥90 days prior to screening, and no history of cardiovascular disease. Participants had normal physical examinations, including vital signs and neurological examination. 12-lead electrocardiogram (ECGs) and serum troponin I had to be normal at screening within 14 days of initial dosing, and serology for hepatitis C antibodies, hepatitis B surface antigen, and human immunodeficiency virus had to be negative. Clinical laboratory test results had to be normal [sodium, total protein, white blood cell count (WBC), hematocrit (Hct), hemoglobin (Hgb), platelet count, aspartate aminotransferase (AST, formerly SGOT), alanine aminotransferase (ALT), and gamma-glutamyl transferase (GGT)], within the upper limit of normal (ULN) and not more than 10% below the lower limit [blood urea nitrogen (BUN), creatinine, total bilirubin, alkaline phosphatase, creatine kinase (CK), and lactate dehydrogenase (LDH)], within 10% of the normal range and not considered to be clinically significant [calcium, chloride, phosphorus, albumin, globulin, cholesterol, triglycerides, and red blood cell count (RBC)], or without clinically significant abnormalities (glucose, potassium, WBC differential, RBC indices, uric acid, and urinalysis). Participants were required to use barrier contraception with spermicide during sexual intercourse during the study and for at least 30 days after the last dose of study drug.

#### Exclusion Criteria

Potential participants were excluded for self-reported chronic use of illicit drugs or other substances of abuse, or for a positive urine drug/alcohol test at screening or any other testing time; for a history or evidence of hepatic, gastrointestinal, renal, respiratory, ophthalmic, cardiovascular, hematologic, endocrine/metabolic, neurologic, immunologic, oncologic, or psychiatric illness or significant abnormalities; or any condition/surgical intervention known to interfere with the absorption, distribution, metabolism, or excretion of drugs. A family history of psychosis, depression, anxiety disorders, or seizures was exclusionary. Potential participants were screened with the major depressive episode, psychotic, and anxiety disorder modules of the Structured Clinical Interview for DSM Disorders IV, with detailed exclusionary criteria. A history of a seizure, head injury, neurosurgery, or brain trauma excluded participation. ECG parameters of heart rate (HR) <45 or >100 bpm, PR intervals of <120 or >220 ms, QRS duration of <70 or >120 ms, or QTcB of >460 ms were exclusionary, as was a history or family history of QT prolongation, arrhythmia, or uncontrolled hypertension. A history of significant alcohol consumption (>3 drinks/day) was disallowed. Participation was excluded for consumption of grapefruit or grapefruit-containing or poppy-seed or quinine-containing substances within 14 days of dosing, or use of any other drug, including prescription and non-prescription medications and herbal supplements, within 30 days prior to dosing (with the exception of acetaminophen, allowed up to 24 h before dosing). Participation in another investigational trial within 45 days of dosing was not permitted. Inability to donate blood, a history of clotting disorders, or donation of >500 mL of blood within 8 weeks of screening was exclusionary. Participation was excluded for unexplained weight loss or gain >10% within 30 days prior to screening, and for allergy to 3-phenyltropane analogs (e.g., brasofensine and tesofensine), heparin, haloperidol, dopamine agonists, or related compounds. Participants were not permitted to perform strenuous exercise within 48 h prior to the screening examination through the final follow-up visit.

### Safety and Tolerability End Points

#### Observational

Orthostatic vital signs (HR, systolic and diastolic blood pressure, and respiratory rate) were obtained pre-dose and at 1, 2, 4, 8, 12, 24, and 48 h post-dose. Body weight was measured at screening, on admissions to the clinic for dosing, and at 7 days following their final dose. Physical examinations of all major organ systems (including neurological examination) were performed at admission and at 48 h post-dose. Adverse events (AEs) were monitored from dosing until 8 days post-dose or resolution, whichever was longer.

#### Electrocardiogram

Six-lead ECG telemetry was monitored from 15 min pre-dose until 8 h post-dose. Supine 12-lead ECGs were obtained pre-dose and at 1, 2, 4, 8, 12, 24, and 48 h post-dose.

#### Clinical Laboratory

Urine for urinalysis (bacteria, bilirubin, blood, glucose, ketones, leukocyte esterase, nitrite, pH, protein, red blood cells, specific gravity, urobilinogen, and WBC) was collected pre-dose and 48 h post-dose. Unfasted blood samples for chemistry (including troponin, albumin, alkaline phosphatase, ALT, AST, BUN, calcium, chloride, CK, creatinine, GGT, globulin, glucose, LDH, phosphorus, potassium, sodium, total bilirubin, total cholesterol, total protein, triglycerides, and uric acid) and hematology (basophils, eosinophils, Hct, Hgb, lymphocytes, RBC indices, monocytes, neutrophils, platelets, RBC, and WBC) were obtained on admission and fasted samples at 48 h post-dose.

#### Mini Mental Status Examination (MMSE)

The Mini Mental Status Examination (MMSE) (Folstein et al., 1975; Malloy et al., 1997) was performed on admission to the clinic and at 4 and 8 h post-dose.

### Pharmacokinetics

Blood samples were obtained at 0.25, 0.5, 1, 1.5, 2, 3, 4, 6, 8, 12, 16, 24, 30, 36, and 48 h post-dose. Cumulative urine specimens were collected over the intervals 0–6, 6–12, 12–24, 24–36, and 36–48 h post-dose.

## Results

### Study Population

The first participants were enrolled on October 2, 2008, and the final participants completed the trial on March 13, 2009. Only 1 of the 22 received only placebo; demographic information is presented in **Table 1**. All were of Hispanic ethnicity; 21 were white and 1 was black/African–American. Mean age was 35 years (range: 20–49) and mean BMI was 27 (range: 23–30). **Table 2** shows the disposition of each participant receiving RTI-336 and placebo by cohort and period. In cohort 1, 10 participants received single escalating doses of 0.3, 1, and 3 mg RTl-336 or placebo; S036 was withdrawn for non-compliance after the first two doses and was not replaced. In cohort 2, 12 participants received single escalating doses of 6, 12, and 20 mg RTI-336 or placebo. S059 and S067 withdrew after the first dose and were replaced by S054 and S077, though per the Medical Monitor S054 was not suitable for dosing in period 2.

**Table 1:** Demographics.

	Parameter	Cohort 1 (*n* = 10)	Cohort 2 (*n* = 12)	All participants (*n* = 22)
Age (years)	Mean (SD)	33.8 (9.4)	35.3 (7)	34.6 (8)
	Median	33	35.5	33
	Range	20–49	25–45	20–49
Gender	Male, *n* (%)	10 (100%)	12 (100%)	22 (100%)
Race	White	9 (90%)	12 (100%)	21 (95.5%)
	Black or African–American	1 (10%)	0 (0%)	1 (4.5%)
Ethnicity	Hispanic, *n* (%)	10 (100%)	12 (100%)	22 (100%)
Height (cm)	Mean (SD)	170 (6.6)	172.4 (5.8)	171.3 (6.2)
	Median	171	172.5	172
	Range	158–179	161–181	158–181
Weight (kg)	Mean (SD)	80.1 (8.9)	77.6 (6.9)	78.7 (7.8)
	Median	80	76.5	76.5
	Range	65–96	67–91	65–96
BMI	Mean (SD)	27.6 (1.8)	26.1 (1.8)	26.8 (1.9)
	Median	28.35	26.35	27.2
	Range	24.2–30	23–28.4	23–30

**Table 2:** Disposition of each participant throughout the trial.

	Subject ID	Period 1	Period 2	Period 3
Cohort 1	S004	0.3 mg	1 mg	Placebo
	S005	0.3 mg	1 mg	3 mg
	S009	0.3 mg	Placebo	3 mg
	S029	0.3 mg	1 mg	3 mg
	S031	Placebo	1 mg	3 mg
	S036	Placebo	1 mg	Not dosed^a^
	S038	0.3 mg	Placebo	3 mg
	S045	0.3 mg	1 mg	3 mg
	S046	0.3 mg	1 mg	3 mg
	S048	0.3 mg	1 mg	Placebo
Cohort 2	S051	6 mg	12 mg	20 mg
	S054^b^	Not dosed	Not dosed	20 mg
	S057	Placebo	12 mg	20 mg
	S059^c^	6 mg	Not dosed	Not dosed
	S064	6 mg	Placebo	Placebo
	S067^c^	6 mg	Not dosed	Not dosed
	S069^d^	6 mg	Not dosed	Not dosed
	S071	6 mg	12 mg	20 mg
	S072	6 mg	12 mg	20 mg
	S075	Placebo	12 mg	20 mg
	S077^e^	Not dosed	Placebo	Placebo
	S078	6 mg	12 mg	20 mg

*^*a*^Withdrawn for non-compliance, not replaced. ^*b*^Replacement subject, not dosed in period 2 due to hold by Medical Monitor for pre-dosing lab value. ^*c*^Withdrew consent in period 2 prior to dosing and replaced. ^*d*^Dosing interrupted for mild CPK increase attributed to heavy lifting. ^*e*^Replacement subject.*

### Safety and Tolerability

One serious adverse event (SAE) and eight AEs occurred during the study (**Table 3**), with no permanent sequelae. The SAE [blood creatine phosphokinase (CK) elevation] was definitely not related to RTI-336; the participant (S004) was a body builder/weight trainer who participated in heavy weight training after his final clinic admission (placebo) but prior to his final follow-up visit, at which his CK was 9,090 U/L (ULN 204). He was kept overnight in the clinic for IV fluid administration to hasten CK clearance, and by day 26 of period 3 his CK had returned to normal (112). The AE experienced by participant S069 was similarly related to his activities and not to study drug. Following 6 mg RTI-336 his CK was 1.2× ULN at the period 1 day 8 visit and dosing was held for period 2; when presenting for dosing in period 3 he again had a CK elevation of 4.5× ULN and stated that he recently had moved heavy furniture. Dosing was again held, and within 1 week his CK had returned to normal (151).

**Table 3:** Adverse events by cohort and period for each participant.

Cohort	Participant	Dose of RTI-336 (mg)				Adverse events
		Period 1	Period 2	Period 3	Cumulative	
1	S004	0.3	1	***0***	1.3	Blood creatine phosphokinase increased^a^
1	S031	0	***1***			Body temperature decreased
	S031			***3***	4	Body temperature decreased, chalazion
1	S038	0.3	0	***3***	3.3	Somnolence^b^
2	S057	***0***	12	20	32	Body temperature decreased^b^
2	S059	***6***	Not dosed	Not dosed	6	Soft tissue inflammation
2	S069	***6***	Not dosed	Not dosed	6	Blood creatine phosphokinase increased
2	S077	Not dosed	***0***	0	0	Headache^b^
2	S078	***6***	12	20	38	Nasal congestion, pharyngolaryngeal pain

*The dose at which the AE occurred is in bold italic text. A dose of “0 mg” is placebo. ^*a*^This event was a serious adverse event. ^*b*^These events were considered by the investigator to be “possibly related” to study drug.*

Overall, five unique participants exposed to RTI-336 and three unique participants who received placebo experienced at least one AE. Three were considered possibly related to study drug: somnolence following 3 mg RTI-336, decreased body temperature following placebo, and headache following placebo. All others were considered definitely not related. There is no obvious dose trend for participants administered RTI-336; when the cumulative dose is considered, the percentage of participants with AEs at cumulative doses less than 6 mg (3 of 10) is similar to that of participants receiving 6 mg or greater (4 of 11).

Two participants had mild, transient elevations of total bilirubin beyond the ULN following RTI-336 dosing. At 1 mg, participant S046 increased from 13.68 on day 0 pre-dose to 22.23 (ULN 20.5) on day 3 post-dose, returning to 8.55 on day 8 post-dose; at 3 mg, the same participant went from 6.84 to 20.52 to 5.13. With 6 mg, participant S069 increased from 10.26 to 25.65 and returned to 10.26. Similar phenomena were not observed with other participants at any dose. No systematic RTI-336 effects were observed on other chemistry or hematology, urinalysis, vital sign, or ECG results. No effects were observed on the MMSE.

Thus, RTI-336 was well-tolerated at single doses of up to 20 mg in this trial. A maximum tolerated dose was not identified.

### Pharmacokinetics

**FIGURE 1:**
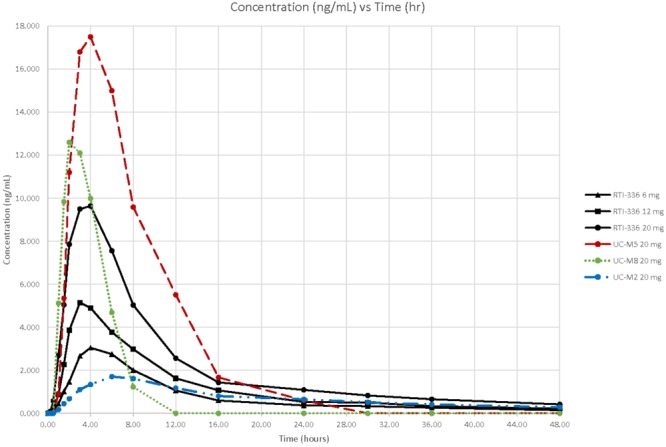
Concentrations of RTI–336 and its metabolites (ng/mL) by time (h) and dose (mg). The solid lines correspond to the plasma RTI-336 concentrations for the three highest, single oral doses of RTI-336 (6, 12, and 20 mg). The broken lines correspond to the plasma concentrations of active metabolites UC-M5, UC-M8, and UC-M2 following a single 20 mg oral dose of RTI-336.

Following oral administration, RTI-336 is readily absorbed. Peak plasma RTI-336 concentrations were observed at about 4 h across 1–20 mg dose levels administered under fasted conditions (**Figure 1** and **Table 4**). The plasma half-life estimates for RTI-336 were similar across the dose levels of 6–20 mg, approximately 18 h, indicating that elimination from plasma follows linear kinetics over this dose range. Plasma drug exposure (AUC_0-inf_) and maximum concentration (C_max_) values increased dose proportionally over the 1–20 mg dose range. Renal excretion was a minor route of elimination, with less than 0.02% of the dose recovered in urine as unchanged RTI-336 over the 48 h post-dose.

**Table 4:** RTI-336 pharmacokinetic parameter estimates following single oral doses of RTI-336.

Dose (mg)	C_max_ (ng/mL)	t_max_ (h)	AUC_0-inf_ (h^∗^ng/mL)	t_1/2_ (h)
1	0.375	4.50	3.80	5.44
3	1.04	3.71	12.3	7.07
6	3.26	4.38	40.4	17.9
12	5.23	3.67	63.1	15.3
20	10.3	3.43	115	18.0

All five known metabolites of RTI-336 (Runyon et al., manuscript in preparation) were measurable in urine, and three were quantifiable in plasma (shown just for the 20 mg RTI-336 dose in **Figure 1**). UC-M5 (a dioxidation and *N*-demethylation product) was most abundant in urine and plasma, followed by UC-M8 (dioxidation product), and then UC-M2 (*N*-demethylation product). The plasma C_max_ for UC-M5 and UC-M8 exceeded that of parent RTI-336, but they had shorter half-lives. UC-M5, UC-M8, and UC-M2 all inhibit dopamine reuptake, with IC_50_ relative potencies of 0.7, 0.4, and 3.5× that of RTI-336 (23 nM).

## Discussion

This phase 1 study in 22 normal men found that RTI-336 was free of significant side effects or toxicity across a dose range of 0.3–20 mg. PK analyses demonstrated half-life estimates averaging approximately 17 h across 6–20 mg doses and relatively slow (about 4 h) attainment of maximum blood levels, which suggests a minimal abuse liability for this DAT reuptake blocker. The metabolic pathways for RTl-336 are primarily to a hydroxymethyl metabolite, followed by oxidation to a carboxyl metabolite, and by *N*-demethylation followed by mono- or di-oxidation. This combination of qualities makes RTI-336 a promising candidate for further testing in stimulant abusers using once daily oral dosing at 20 mg.

The slower onset and an extended duration of action for RTI-336 relative to cocaine should assure less abuse potential than cocaine and hold promise for it as a pharmacotherapy in humans (Samaha and Robinson, 2005; Kimmel et al., 2007). Recent studies of oral amphetamine, which has a faster onset than RTI-336 but shares the same type of extended duration, have been promising in cocaine use disorder patients both with and without adult attention deficit hyperactivity disorder (Grabowski et al., 2001; Levin et al., 2015). The amphetamine dosage needed for effective treatment has been modest but probably is greater than the 20 mg tested in this phase 1 study of RTI-336, based on the mean 50 mg daily dosing of mixed amphetamine salt used in these previous studies. Nevertheless, human laboratory studies using the 20 mg dose of RTI-336 in combination with intravenously administered cocaine should be considered in order to move this potential cocaine pharmacotherapy forward as a once daily treatment. The animal studies support the safety of conducting such studies in humans. Rats which were co-administered RTI-336 with cocaine showed no potentiation of the acute toxicity of cocaine (data on file, RTI). Furthermore, co-administration of RTI-336 with ethanol reduced ethanol-induced incoordination in rota-rod performance and did not increase ethanol-related mortality (data on file, RTI), and co-administration of RTI-336 with morphine did not affect morphine-related analgesia but did increase weight loss (data on file, RTI) as might be expected from potential appetite suppression of this long-acting stimulant.

Preclinical toxicology identified several potential adverse effects that might also have been found in humans. First was central nervous system stimulation with hyperactivity and stereotypic behavior in rats and monkeys, which were acute and reversible with reduced dosing. We showed no such behavioral stimulation in these humans. Second was seizures, which were observed only in one dog at 3 mg/kg at 22 h after the dose and which did not occur in mice at up to 75 mg/kg – 7.5-fold larger than the oral ED_50_ for spontaneous locomotor activity in mice. In our human study the maximum dose was less than 0.3 mg/kg, more than 10-fold below the dose associated with a seizure in dogs. Third were cardiovascular effects with a low potential for prolonging QTc interval. We observed no ECG abnormalities associated with RTI-336 in humans. Fourth was hepatotoxicity, because a 14-day toxicity study in monkeys given 9 mg/kg/day showed increased ALT activity and/or bilirubin and mild-to-moderate diffuse or centrilobular vacuolar degeneration in hepatocytes. In the current human study, participants were 30-fold below this monkey dose. There were two participants who had transient and mild serum total bilirubin elevations (<1.5× ULN and thus Grade 1 by the National Cancer Institute Common Terminology Criteria for Adverse Events, version 4.03) at day 3 post-dose which resolved by day 8 post-dose. In participant S046, 1 mg RTI-336 was followed by an increase to 1.08× ULN and later 3 mg was followed by an increase to 1.001× ULN. In participant S069, the increase following 6 mg RTI-336 was to 1.25× ULN. These are of unclear significance and may have been idiosyncratic reactions. There are no data suggesting a dose response among the other participants.

In summary, the highest dose used in the current study showed excellent safety and was well below the dose that had been associated with any toxicity in animals. Thus, further studies in humans are warranted, particularly concurrent cocaine and RTI-336 administration to test medical safety as well as surrogate markers for efficacy such as attenuation of subjective effects of cocaine.

## Author Contributions

TK, JB, LW, and BW participated in the research design and performed the data analysis. All the authors contributed to the writing of this manuscript.

## Conflict of Interest Statement

TK is the Jay H. Waggoner Chair & Professor of Psychiatry, Pharmacology, Neuroscience and Immunology & Pathology at the Baylor College of Medicine. JB was a full-time employee of RTI Health Solutions at the time the study was conducted and in that capacity also was supported by this grant. LW is a private consultant who wrote the IND application for this study, as well as participating in the analysis and interpretation of the results. BW is a salaried employee of RTI International and was not supported by the grant. There are no other competing financial interests to disclose. The remaining author declares that the research was conducted in the absence of any commercial or financial relationships that could be construed as a potential conflict of interest.
